# Canadian Otolaryngology - Head and Neck Surgery clerkship curricula: evolving toward tomorrow’s learners

**DOI:** 10.1186/1916-0216-42-33

**Published:** 2013-05-03

**Authors:** Kate Kelly, Kevin Fung, Laurie McLean

**Affiliations:** 1 Department of Otolaryngology-Head & Neck Surgery, University of Ottawa, Ottawa, ON, Canada; 2 Department of Otolaryngology-Head & Neck Surgery, Western University, London, ON, Canada

## Abstract

**Background:**

Increasing focus is being placed on Clerkship curriculum design and implementation in light of new undergraduate medical education research and accreditation standards. Canadian Otolaryngology-Head and Neck Surgery (OTOHNS) Clerkship programs are continually but independently evolving towards a common goal of improving Clerkship curriculum.

**Methods:**

An electronic survey was sent to undergraduate OTOHNS directors at all Canadian medical schools (n = 17) examining their Clerkship curricula. Themes included Clerkship format, teaching methods, faculty support and development, program strengths, and barriers.

**Results:**

Survey response rate was 76%. All responding schools had OTOHNS Clerkship programs ranging in type (mandatory, selective or elective) and length (<1 to 4 weeks). Learning modalities varied. Electronic learning tools were identified as increasingly important to curriculum delivery. Common strengths included wide clinical exposure and one-on-one mentoring. Multiple challenges were identified in curriculum implementation and evaluation. All schools expressed interest in developing national standards, objectives and e-learning resources.

**Conclusions:**

Significant variation exists in OTOHNS Clerkship experiences between Canadian medical schools. Many schools perceive barriers of insufficient time, space and curriculum standardization. Interested Canadian OTOHNS educators are eager to collaborate to improve the collective OTOHNS Clerkship experience.

## Background

The current framework for medical education was forged over 100 years ago when Abraham Flexner, commissioned by The Carnegie Foundation for the Advancement of Teaching, published his report, *Medical Education of the United States and Canada: A Report to The Carnegie Foundation for the Advancement of Teaching* (Flexner 1910). The report was an objective evaluation of the quality of North American medical school curricula, and his recommendations provided the basis for the structure of medical education in North America and Europe. Despite immense changes in education and medicine, as well as an improved understanding of teaching and learning through rigorous research, there has astonishingly been little change in medical education over the last century [1].

Much has changed in the medical landscape over the last 100 years, and medicine continues to evolve at an ever-increasing pace. In 2004, The Carnegie Foundation therefore commissioned another study of medical education to reflect the current time. Their site examinations and comprehensive review of current medical education and learning sciences research resulted in four recommendations for the future of medical education:

1. Standardize learning outcomes and individualize learning processes to allow for the integration of new technologies, such as simulation, on-line learning, and mobile learning resources;

2. Integrate formal knowledge with clinical experience;

3. Incorporate habits of inquiry and improvement into medical education at all levels; and

4. Focus on the progressive formation of professional identity [1].

**Figure 1:**
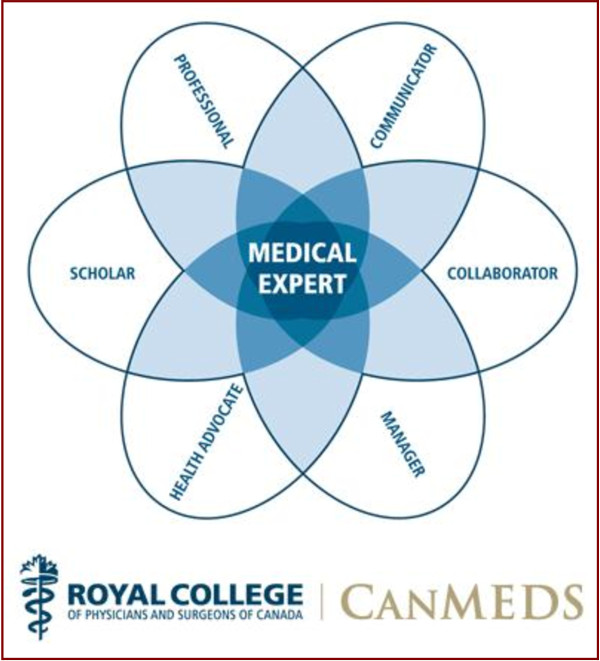
**CanMeds Physician Competency Framework**^**©**^**.**

Canada is also a leader in medical education reform. The Royal College of Physicians and Surgeons of Canada introduced the CanMeds Physician Competency Framework roles of residency education, which has become a leading framework worldwide (Figure 1). More recently, the Association of Faculties of Medicine of Canada (AFMC) released a report entitled “The Future of Medical Education in Canada.” This Health Canada-funded, multi-stakeholder project was a thorough review of undergraduate medical education in Canada. The report released in January 2010 compares society’s present and future needs with Canadian medical curricula and offers ten recommendations and five enabling recommendations to promote excellence in patient care through reform of the medical education system where needed to better prepare the doctors of tomorrow for the century ahead (Table 1) [2].

**Table 1:** FMEC recommendations: a collective vision for MD education

** 10 recommendations**	**5 enabling recommendations**
I: Address Individual and Community Needs	A: Realign Accreditation Standards
II: Enhance Admissions Processes	B: Build Capacity for Change
III: Build on the Scientific Basis of Medicine	C: Increase National Collaboration
IV: Promote Prevention and Public Health	D: Improve the Use of Technology
V: Address the Hidden Curriculum	E: Enhance Faculty Development
VI: Diversify Learning Contexts	
VII: Value Generalism	
VIII: Advance Inter- and Intra- Professional Practice	
IX: Adopt a Competency-Based and Flexible Approach	
X: Foster Medical Leadership	

In light of the Health Canada’s recommendations for medical education reform and the globally changing landscape of medicine, subspecialty undergraduate teaching must also be assessed, and areas of strengths, weaknesses and educational gaps identified. The Canadian Society of Otolaryngology – Head and Neck Surgery (OTOHNS) Undergraduate Medical Education Working Group was formed in 2009 by representatives from OTOHNS departments at all Canadian medical schools to advance OTOHNS undergraduate education across Canada by

1. Creating a national OTOHNS content standards for Canadian medical school graduates;

2. Developing open access, freely available e-learning resources in OTOHNS; and

3. Forming a community of practice of OTOHNS medical educators to develop, assess and disseminate educational OTOHNS resources [3].

In this paper we examine the status of OTOHNS education during the Clerkship or clinical teaching years in Canadian medical schools.

## Objectives

1. To review the current landscape of OTOHNS education at the Clerkship level across all Canadian medical schools.

2. To identify obstacles faced by OTOHNS educators in delivering effective OTOHNS Clerkship training.

3. To determine avenues for improvement of current Clerkship curricula.

## Methods

An electronic survey was sent via Survey Monkey™ to OTOHNS undergraduate directors at each of the 17 Canadian medical schools. The survey consisted of 38 questions, both quantitative and qualitative in nature. Themes of the survey included:

• Clerkship structure and content, including objectives and clinical exposure

• Teaching methods, as well as tools and resources utilized as teaching aids

• Recruitment of and faculty development for physicians in teaching roles

• Innovations, strengths and obstacles identified by the responders

Quantitative data were analyzed using Excel™ and Survey Monkey™. Qualitative responses were reviewed for themes.

## Results

Responses were received from 13 of 17 institutions, a rate of 76%.

1) Structure

**Figure 2:**
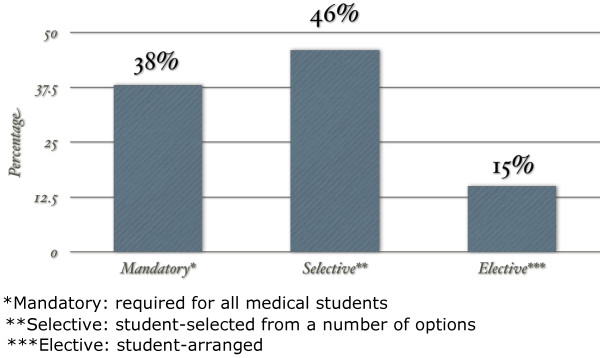
Clerkship structure.

Significant variation exists across Canadian medical schools with respect to Clerkship structure. All responding schools have an OTOHNS Clerkship program. The majority (6/13) offer OTOHNS as a selective rotation only, usually two weeks or more in duration; five schools have mandatory rotations, usually less than one week in duration. Two institutions offer OTOHNS as part of student-arranged elective rotations only (Figure 2).

Formal written learning objectives are in place for 12/13 programs, but the extent to which they are used to guide assessment of competency was unclear from our survey.

2) Content

CanMEDS roles are addressed to varying degrees at each institution, but universally the Medical Expert role is heavily emphasized (64%) (Table 2). Areas of greatest clinical exposure include General OTOHNS (78%), and the subspecialties of Pediatric OTOHNS (70%), Rhinology (78%) and Head & Neck Surgery (70%). All of these are generally at tertiary care centres (Table 3). Only 5/13 institutions consistently offer OTOHNS exposure outside the tertiary care setting.

3) Teaching methods

Teaching opportunities vary widely from school to school, but most commonly include combinations of experience in the ambulatory setting, exposure to the operating theatre, attendance at grand rounds, and provision of learning resources (Table 4). Teaching is mainly achieved through didactic (58%) and one-on-one instruction (75%). Two-thirds of responding schools provide Clerks with OTOHNS-specific learning resources. Of these, e-resources are most common (60%) - these may include websites, power-point modules, apps, podcasts, etc. Other forms of learning resources provided include textbooks (13%) and OTOHNS handbooks specially designed for clerks (38%). One-third of programs offer no specific OTOHNS learning resources to their students.

4) Physician involvement in teaching

On average, each institution reports five to ten or more physicians involved in clinical teaching of clerks, and two to four physicians involved in didactic teaching. Excellence in teaching is recognized in various ways at each institution, and may include combinations of presentation of teaching awards (33%), provision of a stipend (26%), consideration for promotion (40%), consideration for allocation of resources (20%), recognition by department chair at the annual review (47%) or within a departmental newsletter (20%). 20% of responders report no special recognition or remuneration in place for teaching at their institutions. Fewer than half of institutions offer professional development courses in teaching and learning to their OTOHNS physicians (Table 5).

5) Strengths

A number of common themes were identified by responders across all programs with respect to strengths and innovations:

• All programs receive satisfactory (50%) to excellent (50%) feedback from the clerks coming through their services.

• Responders perceive that students are offered wide clinical exposure through ambulatory clinics and in-patient interactions, with a high level of one-on-one mentoring from staff and residents in the clinical setting.

• The development and incorporation of electronic resources as teaching tools is becoming increasingly popular among all institutions polled.

6) Obstacles

A number of obstacles to the effective delivery of OTOHNS education in Clerkship were also identified by the responders, and these generally centred around five main themes:

1. Space

Insufficient teaching space, resulting in a decrease in the independence afforded to clerks when they are forced to follow other learners or staff thus decreasing the opportunity for active learning. Similarly, there are often too many clerks for the number of available teachers, again decreasing teaching and learning time.

2. Time

Lack of dedicated teaching time, both due to short rotation lengths and busy clinics.

3. Teaching Strategies

Common obstacles to effective teaching include wide variation in clinical exposure even within institutions, insufficient available teaching tools such as online videos, e-modules, video scopes (otoscope, laryngoscope) and monitors.

4. Faculty development

Insufficient knowledge and training among physician teachers of new effective teaching strategies.

5. Teacher Support

Lack of strategies that encourage dedicated physician teaching which include but are not limited to recognition, training, compensation and promotion (Table 6).

**Table 2:** Q.10 - How well are the following CanMEDs roles taught during the OTOHNS clerkship at your institution?

	**Not at all (%)**	**Somewhat (%)**	**Well (%)**
**Medical expert**	**0.0**	**35.7**	**64.3**
Collaborator	7.1	57.1	35.7
Communicator	7.1	50.0	42.8
Scholar	7.1	64.3	28.6
Manager	28.6	42.9	28.6
Professional	14.3	50.0	35.7
Health advocate	14.3	57.1	28.6

**Table 3:** Q.9 - Are your students exposed to the following in the UME clerkship rotation

	**Always**	**Sometimes**	**Never**
**General**	**78.6**	21.4	0.0
Laryngology	38.5	53.8	7.7
**Pediatric**	**69.8**	30.2	0.0
**Rhinology**	**69.8**	30.2	0.0
Otology	46.2	53.8	0.0
Facial Plastics	0.0	92.3	7.7
**Head & Neck**	**76.9**	23.1	0.0
Reconstructive	23.1	61.5	15.4
Audiology/Vestibular Testing	14.3	78.6	7.1
Speech Language Pathology	0.0	64.3	35.7

**Table 4:** Q.5 - What components are included in your standard OTOHNS clerkship curriculum?

	**%**
Ambulatory clinic	86.7
Operating theatres	80.0
On call	20.0
Audiology	6.7
Speech language pathology	0.0
Departmental grand rounds	40.0
Dedicated UME seminars	46.7
Simulation	6.7
Mandatory e-learning modules	13.3
Recommended e-learning modules	40.0

**Table 5:** Q.30 and Q.31 - Does your department and/or university provide faculty development in teaching and learning (mandatory or non-mandatory) for your OTOHNS MDs?

	**Department (%)**	**University (%)**
Yes	14.3	66.7
No	50.0	13.3
Unsure	35.7	20.0

**Table 6:** Q.27 - What are significant barriers in the OTOHNS Clerkship rotation at your institution?

	**%**
Limited time with students	71.4
Limited faculty involvement	28.6
Limited space/infrastructure	42.9
Learner: teacher ratio too high	50.0
Technology without teaching elements (i.e. scopes without video)	64.3
Lack of recognition for MDs with UME involvement	42.9
Lack of innovative teaching tools or their awareness	35.7

## Discussion

The landscape of undergraduate medical education in Canada is changing resulting in an immense opportunity for educators. Taken together, the detailed responses received from our survey highlight a number of directions for future improvement of OTOHNS Clerkship medical education on a national scale.

### Create usable learning objectives

While nearly all institutions polled had formal written learning objectives, a review of these objectives revealed they were both extensive and exhaustive. The creation of usable learning objectives is a paramount first step to guide structure and consistency both within and between Canadian medical schools, as well as develop a national standard to which all medical students can be held accountable. In the creation of Clerkship-specific learning objectives, the objectives should be specific to the clinical teaching years, with the aim to provide a foundation for further learning in residency programs and continuing professional development, not to train OTOHNS subspecialists. To be effective, objectives should include an action or behaviour statement, a condition and a standard. Similarly, the objective should focus on core competencies that can be measured and demonstrated within the duration of the rotation. Finally, objectives should form the basis of evaluation and assessment of medical students.

### Maximize learning time

The complaint of insufficient teaching time is universal among all specialties. With so many competing interests and the inherent limitations of space, budget and person-power, significant increases in teaching time are unlikely to be realized. Still, increased teaching *efficiency* may be achieved by maximizing the learning opportunities already in place.

**Figure 3:**
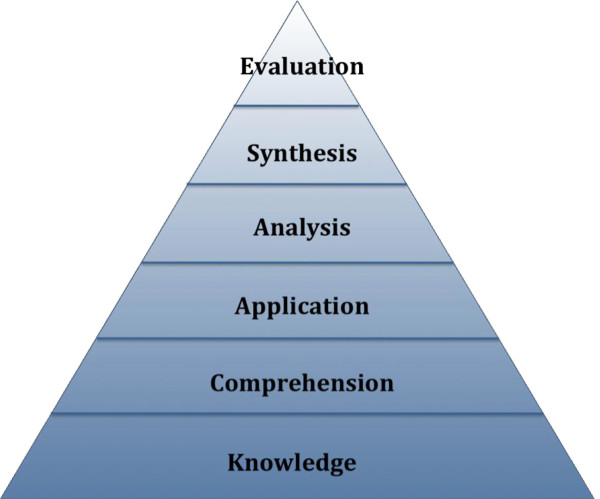
Bloom’s Hierarchy of Cognition.

In 1956 Benjamin Bloom and a committee of educators published a framework for categorizing cognitive domains, essentially mapping out a hierarchy of learning progression (Figure 3) [4]. Although it has undergone some minor revisions over the years, Bloom’s taxonomy remains the foundation for understanding the advancement of thought processes as learners transfer learned knowledge to actual practice [4]. Bloom’s hierarchy of domains include:

• Knowledge

recalling information

• Comprehension

grasping the meaning of concepts

• Application

using a previously learned concept in a new situation

• Analysis

identifying relationships among parts of a concept

• Synthesis

putting different concepts together to form a new product

• Evaluation

judging the results [4].

As suggested by the taxonomy, greater teaching efficiency may be achieved by focusing on the higher orders of learning such as application, synthesis and evaluation rather than knowledge acquisition and comprehension. Providing approved supplemental resources to students for self-study may improve learning efficiency by building foundational knowledge upon which further expansion can occur in the clinical setting. For example, advanced preparation by the student in select problem based learning e-modules prior to attendance at clinic may allow for improved practical application of knowledge by the student and “expert” discussion time with staff preceptors, thus resulting in higher level learning according to Bloom’s Taxonomy (e.g. application, analysis and synthesis) . For in-clinic learning, the adoption of the “1-minute preceptor” model might encourage quick synthesis of specific data and knowledge application to real patient scenarios. In addition, these higher orders of learning may be made the focus of specific learning activities, such as electronic self-study learning modules, as discussed below.

The responses to our survey demonstrate that while many schools make use of independent learning resources, up to one-third of Canadian institutions do not provide their students with OTOHNS-specific resources for self-study. For today’s information-hungry population of learners, this may represent a significant missed opportunity for efficient advancement of learning. Tapping into the technology-based mainstream for communication and information dissemination may represent a highly effective way of improving access to students and increasing teaching efficiency [5].

### E-resource development

The present “millennial generation” of medical students has grown up in a digital world, one with instant access to information and where the language of technology is the native tongue. Attention spans may be shorter, multi-tasking is often the norm, and individuals are seemingly always connected – to data, and to each other.

As the Internet has made access to information almost instantaneous, the wealth of resources available to medical students today is rapidly expanding. In North America, textbooks and notebooks are being replaced by tablets and smartphones. Many medical schools offer web-based teaching resources, but the format, availability, style and content of these vary widely [6]. When created using pedagogical principles, e-learning resources complement the Constructivist Theory of Learning and result in large positive effects in knowledge outcomes, satisfaction, skills and learner behaviours [7,8]. Well-designed e-learning resources naturally lend themselves to interactive learning applications, are easily accessible by students for self-study, can be utilized by clinical clerks who do not complete mandatory OTOHNS rotations, and can be pooled and shared among institutions across the country. In addition, content can be viewed and reviewed at the convenience of the learner for reinforcement [6,8].

As the popularity of portable media devices and smartphones has grown, so has the use of podcasts. A survey of second-year medical students at the University of Leeds revealed 75% owned a portable digital media player and 90% listened to podcasts on most days [6]. Video podcasting has become an increasingly popular tool for medical education as content can be viewed anytime, anywhere, and multiple times. Furthermore, content can be peer-reviewed to ensure evidence-based content and clinical accuracy, in contrast to information available from many publicly accessible web-based resources. Currently, most podcasts for undergraduate students are in the form of entire lectures that have been recorded and made accessible online [6].

However, perhaps there are more innovative e-learning tools that can be created [9]. As many authors on the subject argue, teaching on the web involves more that putting together a colorful webpage or jazzy power-point presentation; it must also employ principles of effective learning [10,11]. For example, Narula et al. of McMaster University in 2012 developed a new strategy for e-teaching tools entitled “5-Minute Medicine (5MM)” which have yielded promising results in Internal Medicine instruction [6]. The 5MM video podcasts are unique in that they are short podcasts related to specific high-yield topics, focusing on core objectives [6]. Research has suggested podcasts should be no longer than 15 minutes for maximum attention and information retention. A high proportion of students trialing the 5MM model reported that the 5MM video podcasts were effective learning tools, appropriate for Canadian clinical clerks, and time-efficient as compared to conventionally used resources, both print- and web-based. The vast majority of clerks selected the 5MM videos as their preferred learning resource [6].

The 5MM model is just one example of the successful application of technology to improve learning efficiency. Many OTOHNS educators across the country have already begun individually to create various e-learning resources for the Clerks at their institutions [12,13]. Infrastructure is a key element in preparing for the changing role of instructional technologies in medical education [14]. OTOHNS UME in Canada is fortunate to have received funding and leadership by the Canadian Society of OTOHNS to enhance electronic infrastructure and in this age of web-based knowledge sharing, the development of a national repository of clinically accurate, evidence-based and pedagogically sound electronic learning resources specific to OTOHNS may be a powerful way to pool these resources and standardize learning from coast to coast.

### Distributed education

A core focus of the new FMEC recommendations is increased emphasis on generalist and community medicine [2]. Interestingly, this call for a shift toward generalism is occurring at the same time as physicians in academic and tertiary care centres find themselves overwhelmed with the number of students they are expected to teach while carrying out their clinical duties. This combination may result in an interesting opportunity: a possible solution to both issues may be to increase involvement of community physicians in teaching roles through distributed medical education (DME). Less than 1% of medical care is delivered in tertiary care hospitals, yet for many medical students this remains the major site of their medical education, a reality that is reflected by the responses to our survey [15]. In recent years a number of authors have extolled the advantages of Community Medical Education, including:

• Greater exposure of students to practice settings more representative of most typical medical practices;

• Access to a wider variety of patients, with more opportunity to develop and practice clinical skills across a broader continuum of care; and

• Better access for communities to the “next generation” physician workforce and thus better able to address local physician shortages through increased recruitment and retention of medical graduates [15].

But increased community exposure requires increased participation by community physicians in medical education. In order for community physician recruitment for teaching to be successful, there must also be benefit to the community Otolaryngologist. Possible benefits might include:

• Offering incentive and/or compensation, which may take the form of allotment of funds to allow increased dedicated teaching time when students are present in their clinical practices

• Providing access to additional resources through the academic centres and affiliated universities

• Providing continuing professional development (CPD) recognition for teaching activities

• Implementing recruitment events to build interest and demonstrate to the community physician why participation in undergraduate medical education is important: (i.e., to promote community practice, to encourage students to return to community practice, to fill needs in underserviced areas, and to contribute to the formation of the next generation of physicians);

• Offering physician-educator training sessions on such topics as effective clinical teaching, teaching in the office setting, etc.; and

• Having realistic expectations for involvement so as to make the commitment enjoyable rather than taxing to the community physician’s time, space and budget [15].

One of the universal strengths lauded by survey respondents is the high level of one-on-one mentoring from staff and residents in the clinical setting, which is directly reflected by the high percentages of student time spent with OTOHNS physicians versus other allied health professionals during their clinical rotations (Table 3). However, the focus of the FMEC guidelines on distributed education also encourages increased inter- and intraprofessional involvement [2]. In the OTOHNS clerkship, clinical exposure of clerks to the other health allied services integral to the provision of good OTOHNS care – including audiology, speech-language pathology, etc. – may serve to meet the common goals of advancing interprofessional education and collaboration, while at the same time distributing OTOHNS clerkship education among more potential teaching sites and educators.

## Conclusion

Medicine is advancing at an increasing pace; greater depth and breadth of medical education research is helping us to better understand the teacher and the learner; and society is changing. This trifold situation has necessitated the evolution of medical education. This landscape analysis can act as a OTOHNS needs assessment, identifying strengths, development needs and gaps. By creating effective national learning objectives; focusing on higher orders of learning to improve learning efficiency; sharing critically reviewed electronic learning resources among institutions; and increasing physician recruitment and community physician involvement in teaching roles, the national OTOHNS undergraduate curriculum may evolve in tandem, both to keep pace with international standards and to meet the new requirements as put forth by Health Canada and the Royal College of Physicians and Surgeons. This study represents the first step on a long road to evolving national Clerkship OTOHNS education, and highlights many exciting possibilities for OTOHNS educators and the CSOHNS UME Working Group to further explore.

## Competing interests

The authors declare that they have no competing interests.

## Authors’ contributions

LM and KF are members of the Canadian Society of Otolaryngology - Head & Neck Surgery Undergraduate Medical Education Working Group. LM and KK created and distributed the survey, collated the results and assisted with the writing of the manuscript. KK analyzed the data, drafted the manuscript, and presented the results at the Canadian Society of Otolaryngology 2012 annual meeting. All authors read and approved the final manuscript.
